# Effects of cysteine addition to low-fishmeal diets on the growth, anti-oxidative stress, intestine immunity, and *Streptococcus agalactiae* resistance in juvenile golden pompano (*Trachinotus ovatus*)

**DOI:** 10.3389/fimmu.2022.1066936

**Published:** 2022-11-17

**Authors:** Jia-Xing Liu, Ke-Cheng Zhu, Hua-Yang Guo, Bao-Suo Liu, Nan Zhang, Dian-Chang Zhang

**Affiliations:** ^1^ Key Laboratory of South China Sea Fishery Resources Exploitation and Utilization, Ministry of Agriculture and Rural Affairs, South China Sea Fisheries Research Institute, Chinese Academy of Fishery Sciences, Guangzhou, China; ^2^ College of Fisheries, Dalian Ocean University, Dalian, China; ^3^ Sanya Tropical Fisheries Research Institute, Sanya, China; ^4^ Guangdong Provincial Engineer Technology Research Center of Marine Biological Seed Industry, Guangzhou, China

**Keywords:** *Trachinotus ovatus*, cysteine, intestine immunity, growth, antioxidant capacity, *Streptococcus agalactiae*

## Abstract

As the precursor of taurine, cysteine serves physiological functions, such as anti-oxidative stress and immune improvement. Investigation of cysteine and its derivatives has made positive progress in avian and mammalian species, yet the study and application of cysteine in aquatic animals are relatively rare. Therefore, we evaluated the effects of supplementing a low-fishmeal diet with various levels of cysteine on the growth, antioxidant capacity, intestine immunity, and resistance against *Streptococcus agalactiae* of the juvenile golden pompano (*Trachinotus ovatus*). According to our study, exogenous supplementation with 0.6-1.2% cysteine greatly increased the final body weight (FBW) and specific growth rate (SGR) of golden pompano compared to the control group. Under the present conditions, the optimum dietary cysteine supplementation level for golden pompano was 0.91% based on the polynomial regression analysis of SGR. Meanwhile, we found that the Nrf2/Keap1/HO-1 signaling pathway was notably upregulated with the increase of exogenous cysteine, which increased antioxidant enzyme activity in serum and gene expression in the intestine and reduced the level of reactive oxygen species (ROS) in the serum of golden pompano. In addition, morphological analysis of the midgut demonstrated that exogenous cysteine improved muscle thickness and villi length, which suggested that the physical barrier of the intestine was greatly strengthened by cysteine. Moreover, cysteine increased the diversity and relative abundance of the intestinal flora of golden pompano. Cysteine suppressed intestinal NF-κB/IKK/IκB signaling and pro-inflammatory cytokine mRNA levels. Conversely, intestinal anti-inflammatory cytokine gene expression and serum immune parameters were upregulated with the supplementary volume of cysteine and improved intestine immunity. Further, exogenous cysteine supplementation greatly reduced the mortality rate of golden pompano challenged with *S. agalactiae*. In general, our findings provide more valuable information and new insights into the rational use of cysteine in the culture of healthy aquatic animals.

## Introduction

As a major concern of the global aquaculture industry, the price of fishmeal has markedly increased over the past years, making the identification of fishmeal substitutes an urgent matter (1–3). However, previous studies indicate that neither plant protein, such as soybean meal (1, 2), rapeseed protein (3), and corn gluten meal (1, 4), nor animal proteins, such as insect meal (5), poultry by-product meal (1), and chicken meal (6), which are closer to fish meal in terms of nutrient composition, can completely replace fish meal in the diet of aquatic animals. As good sources of protein, these alternatives may have advantages in terms of their price, and small quantities required, however, the role of fishmeal as a bottleneck in aquafeeds seems impenetrable (7–9). Studies have shown that if the proportion of fishmeal is markedly reduced, it can cause a change in the intestinal microbiota of aquatic animals (10, 11), leading to a decline in growth performance (11, 12). The presence of abundant anti-nutritional factors in their diet can elicit oxidative stress in aquatic animals (9, 11). This typically results in structural damage to the intestinal tract (12) and ultimately leads to a decline in intestine immunity (8, 12–15), reducing the ability of aquatic animals to cope with external pathogen infection and, thus, leading to death. Therefore, considering fishmeal scarcity and the lack of an adequate substitution, it is important to explore novel ways to reduce the proportion of fishmeal in aquatic animal diets while maintaining growth, reducing oxidative stress, and enhancing intestine immunity.

Previous studies have shown that appropriate inclusion of functional supplements in aquatic diets can effectively mitigate the adverse effects of low-fishmeal diets on fish (11, 16). Taurine is a functional amino acid that promotes fish growth and immunity, and its levels are correlated with fish health (17–19). The primary source of dietary taurine is fishmeal, thus reducing the proportion of fishmeal in the diet of fish will inevitably decrease dietary taurine content and reduce growth performance and intestine immunity (16, 19).

Cysteine, the precursor of taurine in living organisms (20, 21), has important biological functions, including antioxidative (22) and immunity (23) enhancing effects. Research on the functions of cysteine and its derivatives in mammals, such as rat (22, 24), pig (25–27), and sheep (28, 29), has produced considerable advances; however, respective research and applications in aquatic animals are relatively scarce. Studies in rats have indicated that cysteine as a dietary additive improves resistance to oxidative stress associated with high sucrose levels (22). Moreover, the potential of cysteine to enhance intestine immunity may be related to its resistance to oxidative stress and defense against inflammatory responses (24). For example, cysteine may protect the physical barrier of piglet intestines through the Nrf2 and NF-κB pathways, increasing the length of intestinal villi, and protecting intestinal structure and functioning (25).

The intestine immunity system, consisting of physical barriers, intestinal flora, and immune barriers, is essential for organismic health (30), and cysteine has been shown to improve intestine immunity. For example, dietary supplementation with 0.4% cysteine can increase the quantity and diversity of intestinal microorganisms in gestating sows (31). In addition, cysteine can revive the intestine immunity system in pigs by downregulating the mRNA levels of pro-inflammatory cytokines and improving the local inflammatory response in the intestine (27). Moreover, cysteine supplementation may, to some extent, protect broiler chickens infected with Eimeria from intestinal lesions (32).

The golden pompano (*Trachinotus ovatus*) is one of the predominant aquaculture fish in China, however, the effects and mechanisms of cysteine on the intestine immunity of golden pompano are not entirely clear. Theoretically, satisfying the 42% dietary protein level of golden pompano with Peruvian fishmeal, containing 67% protein, would require a diet with a minimum of 60% fishmeal, which would inevitably increase the cost of farming given the current shortage of fishmeal resources (33). Nevertheless, diets with lower fishmeal content tend to reduce the immune capacity of fish (8, 9). Infectious diseases caused by *Streptococcus agalactiae* frequently broke out in recent years in large-scale cultures of marine fish due to excessive farming density and increasing environmental pollution (34). Therefore, we explored the effects of cysteine-supplemented low-fishmeal diets (containing 20% fishmeal) on growth performance, intestine immunity, and resistance against *S. agalactiae* in juvenile golden pompano in order to provide further insights into the mechanism of cysteine regulation of the intestine immunity of fish and to identify a solution for future usage of low-fishmeal diets.

## Materials and methods

### Ethical statement

All experiments in this study were conducted in accordance with the regulations and guidelines established and approved by the Animal Care and Use Committee of the South China Sea Fisheries Research Institute of the Chinese Academy of Fishery Sciences (No. SCSFRI96-253).

### Experimental diets

Experimental diets of golden pompano were supplemented with food-grade cysteine (99.99% purity, Zhejiang Yi Nuo Biotechnology Co. Ltd., Wenzhou, China). The remaining ingredients were supplied by Guangzhou Nutriera Biotechnology Co. Ltd. (Guangzhou, China); **Table 1** shows dietary formulations and nutritional levels. We produced five diets with equal nitrogen and lipid, based on the nutritional requirement of 42% protein for golden pompano (35). We mixed protein sources to avoid one of them reducing palatability; animal protein (fish meal and chicken meal) and plant protein (soybean protein concentrate, fermented soybean meal, and corn protein meal) were used as the base protein sources for golden pompano diets. Fish oil and soybean oil were used as sources of lipids. High gluten flour provided relatively little protein and fat and was used as a gamete to achieve the same quality in all diets (33). We set the amount of cysteine added to the diet at 0.00%, 0.30%, 0.60%, 0.90%, and 1.20% for groups C0 (control), C1, C2, C3, and C4 respectively, with reference to the amount of cysteine added to the *Paralichthys olivaceus* diet (36). In brief, the first step was to grind all solid ingredients separately until they could pass through a 40-mesh screen. Secondly, the ground ingredients were mixed at the proportions shown in **Table 1**; oil and water were added in batches during mixing, and each batch of diets was mixed for 30 min to achieve homogenization. The mixture was then placed in an extruder (Valva Machinery Co., Ltd., Guangzhou, China), and the diets were transformed into three sizes of spherical pellets (1, 2, and 3 mm diameter) to suit the growth state of the fish (37). Finally, the diets were placed in a drying oven at 45°C until the moisture content was reduced to approximately 10% (38); thereafter, the pellets were stored 4°C. **Table 2** shows the amino acid composition of each diet.

**Table 1:** Formulation and nutrition level of the experimental diets (% dry matter basis).

Parameters	Group				
	C0	C1	C2	C3	C4
Ingredients (%)					
Fishmeal ^a^	20.00	20.00	20.00	20.00	20.00
Chicken meal ^a^	10.00	10.00	10.00	10.00	10.00
Soy protein concentrate ^a^	10.00	10.00	10.00	10.00	10.00
Squid paste	5.00	5.00	5.00	5.00	5.00
Soybean meal ^a^	12.00	12.00	12.00	12.00	12.00
Fermented soybean meal ^a^	5.00	5.00	5.00	5.00	5.00
Corn gluten meal ^a^	6.00	6.00	6.00	6.00	6.00
High gluten flour ^a^	18.37	18.07	17.77	17.47	17.17
Fish oil ^a^	6.00	6.00	6.00	6.00	6.00
Soybean oil ^a^	3.00	3.00	3.00	3.00	3.00
Ca(H_2_PO_4_)_2_ ^a^	1.50	1.50	1.50	1.50	1.50
Choline chloride ^a^	0.30	0.30	0.30	0.30	0.30
Vitamin mix ^a,b^	1.00	1.00	1.00	1.00	1.00
Mineral mix ^a,c^	1.00	1.00	1.00	1.00	1.00
L-lysine monohydrochloride ^a^	0.50	0.50	0.50	0.50	0.50
DL-Methionine ^a^	0.20	0.20	0.20	0.20	0.20
Threonine ^a^	0.10	0.10	0.10	0.10	0.10
Ethoxyquin ^a^	0.03	0.03	0.03	0.03	0.03
Cysteine ^a^	0.00	0.30	0.60	0.90	1.20
Nutrition level ^d^					
Crude Protein (% dry matter)	42.79	42.74	42.69	42.63	42.58
Crude Lipid (% dry matter)	13.42	13.40	13.38	13.37	13.35
Moisture (% dry matter)	10.15	10.76	11.24	10.98	11.32
Ash (% dry matter)	8.53	8.65	8.33	8.71	8.39
Cysteine	0.52	0.82	1.13	1.45	1.84

a Ingredients are provided by Guangzhou Nutriera Biotechnology Co., Ltd. and Zhejiang Yi Nuo Biotechnology Co. Ltd.

b Vitamin mix provides the following (Per kilogram content): vitamin A (8×106 IU), vitamin D3 (2×106 IU), vitamin E 40 000 mg, vitamin B 17 000 mg, vitamin B6 12 000 mg, vitamin B12 100 mg, vitamin K3 10 000 mg, D-pantothenic acid 35 000 mg, folic acid 1 000 mg, nicotinamide 90 000 mg, Biotin 200 mg, inositol 80 000 mg.

c Mineral provides the following (Per kilogram content): Fe 10 000 mg, Cu 1 200 mg, Zn 7 000 mg, Mn 5 500 mg, Co 250 mg, I2 250 mg, Se 50 mg, K 60 000 mg, Na 24 000 mg, Mg 60 000 mg

d Nutrition level is measured.

**Table 2:** Amino acid composition of the experimental diets(g·100g^−1^).

Parameters	Group				
	C0	C1	C2	C3	C4
Aspartic acid	4.35	4.39	4.24	4.31	4.45
Threonine	1.90	2.02	2.11	1.91	2.02
Serine	2.04	2.05	2.04	2.12	2.05
Glutamic acid	8.41	8.66	8.69	8.36	8.62
Glycine	3.04	3.1	3.02	2.98	3.08
Alanine	3.06	3.09	3.06	2.95	3.07
Proline	3.21	3.13	3.04	3.16	3.13
Valine	2.13	2.11	2.07	2.12	2.11
Methionine	1.18	1.15	1.12	1.31	1.12
Isoleucine	1.77	1.84	1.84	1.82	1.80
Leucine	4.42	4.50	4.42	4.41	4.39
Tyrosine	1.27	1.30	1.31	1.29	1.22
Phenylalanine	2.24	2.32	2.25	2.37	2.25
Lysine	3.54	3.43	3.50	3.54	3.50
Histidine	1.11	1.13	1.12	1.07	1.15
Arginine	3.15	3.10	3.14	3.15	3.11
Cysteine	0.52	0.82	1.13	1.45	1.84

### Experimental procedure

To reproduce the large-scale culture environment of golden pompano, we used offshore cages (1.00 × 1.00 × 1.50 m) in Longgang District, Shenzhen, China. *S. agalactiae* was extracted from golden pompano and tested for its pathogenicity. The fish used for this experiment were selected from juvenile golden pompano bred in our laboratory throughout the year. Before the experiment, we separated 1,050 fish (10.05 ± 0.05 g) into 15 cages (five diets, three cages per diet), with 70 fish per cage. Since fish mortality is inevitable during the feeding process, we set the number of fish per cage to 70 to ensure that there would also be enough fish for subsequent challenge experiments. The fish were then fed the C0 diet without exogenous cysteine for one week to allow the fish to adjust to the experimental conditions. During the experiment, we fed the fish their respective diet at 8:00, 10:00, 14:00, and 16:00 each day, until the fish stopped eating. During the eight-week feeding period, feed intake status and hydrographic information at sea were observed and recorded daily. During the test period, the water conditions were maintained as follows: temperature at 28–32°C, pH at 7.4–8.3, salinity at 34–36 ‰ and dissolved oxygen > 6.0 mg/L.

### Sample collection

At the end of the feeding experiment, the fish were fasted for 24 h, after which the fish were weighed and counted in each cage. Nine fish per cage were randomly chosen, and anesthetized with eugenol (100–200 mg/L; Shanghai Medical Devices Co., Ltd., Shanghai, China). Three fish per cage were transferred to -20°C after rapid freezing with liquid nitrogen for organism composition analysis. From three other individuals per cage, we collected blood and centrifuged it to obtain serum for measuring anti-oxidative stress and immunological parameters. After serum collection, the fish were dissected, and the intestines were collected for RNA extraction and gene expression analysis. The intestinal contents were removed and stored at -80°C for intestinal flora analysis. The midguts of the remaining three fish per cage were collected and preserved in 4% paraformaldehyde solution for histological analysis.

### Growth performance

Growth performance-related parameters were calculated according to the following equations:


WGR (%) = 100 × (FBW − IBW) / IBW

SGR (% /day) = 100 × (ln FBW −ln IBW) /NOD

FCR (%) = 100 ×DDI /NWG

$$\text{CF (g }/{\text{cm}}^{3})\;=\;100\;\times \text{ FBW }/{\text{FBL}}^{3}$$

SR (%) = 100 × FFN /30

HSI (%)= 100 ×LW /FBW

VSI (%)= 100 ×VW /FBW

FI (% /day) = 100 ×DDI / [(IBW + FBW) /2] /NOD

### Serum biochemical and immunological parameter

To investigate the effects of exogenous dietary cysteine on the anti-oxidative stress capacity and immunity in juvenile golden pompano, we examined the relevant parameters in serum. For antioxidant indices, we determined the levels of MDA, ROS, and the activities of antioxidant enzymes, such as T-AOC, CAT, GSH-PX, and SOD. Serum immunological indicators included LZM, complement 3, complement 4, and immunoglobulins (IgA, IgG, and IgM).

### Midgut histological examination

In accordance with a previous study (39), we stained the fish midgut with hematoxylin and eosin to observe effects of exogenous cysteine on the intestinal physical barrier. Briefly, the midguts were stored in a 4% paraformaldehyde solution for 24 h. We used ethanol to gradually eliminate the moisture. The midguts were then transferred to paraffin. When the paraffin was solidified, it was cut into 5-μm-thick slices using a microtome. Next, the slices were placed on slides and were stained with hematoxylin and eosin for nucleus and cytoplasm staining. Finally, we completely scanned the midgut sections using a light microscope (Leica, Wetzlar, Germany) with 200-fold magnification, divided the scans into eight equal parts, and randomly measured the length of intact intestinal villi, muscle thickness, and the number of goblet cells per villus in each part using Image-Pro Plus 6.0 software (National Institutes of Health, Bethesda, USA). The data were imported into GraphPad Prism 8 software (San Diego, California, USA) to examine differences and to draw graphs.

### 16S rDNA high-throughput sequencing of the intestinal flora

According to previous studies (39, 40), total DNA of intestinal bacteria was obtained using a DNA extraction kit (TIANGEN BIOTECH Co., Ltd., Beijing, China), DNA integrity was assessed using 1% agarose gel electrophoresis, and concentration was measured using a Nanodrop 2000 device (Thermo Fisher Scientific, Waltham, MA, USA). The DNA samples were diluted to 1 ng·μL^-1^ using sterile water, and PCR amplification of the bacterial 16S rDNA V3–V4 variable region was performed using specific primers. The primers 341F (5′-CCTAYGGGRBGCASCAG-3′) and 806R (5′-GGACTACNNGGGTATCTAAT-3′) were used to amplify the V3–V4 variable region. The reaction system and procedure for PCR amplification were used as described previously (39). PCR products were recovered using an AxyPrepDNA Gel Recovery Kit (AXYGEN Inc., California, USA) for gel cutting, Tris HCl elution, and 2% agarose electrophoresis for detection. The PCR products were quantified using a QuantiFluor™-ST Blue Fluorescence Quantification System (Promega Corporation, Madison, Wisconsin, USA), based on the preliminary quantification results of electrophoresis. Paired-end Illumina libraries were then constructed by mixing the corresponding ratios according to the sequencing volume requirement of each sample. Paired-end reads obtained using an Illumina sequencing platform (Illumina, San Diego, CA, USA) were first spliced according to the overlapping relationship, the sequences were quality-controlled and filtered, and the samples were differentiated and then subjected to operational taxonomic unit (OTU) clustering and clustering-based taxonomy analyses. Various diversity indices were produced based on the OTU clustering analysis. Abundance, alpha diversity (Chao1 index, Shannon index, and Simpson index), beta diversity (principal component analysis [PCA], UniFrac-based principal coordinate analysis [Pcoa], and UniFrac-based non-metric multi-dimensional scaling [NMDS] analysis), and linear discriminatory analysis were performed on OTUs to obtain information on species richness and evenness within the samples. Dilution curves were plotted using R software (V3.6.3, University of Auckland, New Zealand.) (36).

### Quantitative real-time PCR

To further examine the modulatory effects of dietary cysteine on the growth, anti-oxidative stress, and intestine immunity of golden pompano, we assayed the expression of several genes. For the antioxidant stress capacity, we measured mRNA levels of antioxidant enzymes (*CAT*, *GSH-PX*, and *SOD*), critical factors of the Nrf2 pathway (*Nrf2* and *Keap-1*), and *HO-1* in the intestine of golden pompano. *EF-1α* was used as the internal reference gene (41). All primer sources are listed in **Table 3**.

**Table 3:** qPCR primer sequences.

Primers	Forward primer sequences (5′-3′)	Reverse primer sequences (5′-3′)	Source
*CAT*	GGATGGACAGCCTTCAAGTTCTCG	TGGACCGTTACAACAGTGCAGATG	Liu et al. (41)
*SOD*	CCTCATCCCCCTGCTTGGTA	CCAGGGAGGGATGAGAGGTG	Liu et al. (41)
*GSH-PX*	GCTGAGAGGCTGGTGCAAGTG	TTCAAGCGTTACAGCAGGAGGTTC	Liu et al. (41)
*HO-1*	AGAAGATTCAGACAGCAGCAGAACAG	TCATACAGCGAGCACAGGAGGAG	Xie et al. (42)
*Nrf2*	TTGCCTGGACACAACTGCTGTTAC	TCTGTGACGGTGGCAGTGGAC	Liu et al. (43)
*Keap-1*	CAGATAGACAGCGTGGTGAAGGC	GACAGTGAGACAGGTTGAAGAACTCC	Liu et al. (43)
*IL-1β*	CGGACTCGAACGTGGTCACATTC	AATATGGAAGGCAACCGTGCTCAG	Liu et al. (41)
*IL-8*	CCGATCAACAGGGACTTCAA	GAGGACCGAGGGTTCAGACAG	Zhang et al. (44)
*IL-10*	AGTCAGTCTCCACCCCCATCTT	GCCCACTGGAGTTCAGATGCT	Zhang et al. (44)
*TNF-α*	GCTCCTCACCCACACCATCA	CCAAAGTAGACCTGCCCAGACT	Liu et al. (41)
*NF-κB*	CGTGAGGTCAGCGAGCCAATG	ATGTGCCGTCTATCTTGTGGAATGG	Liu et al. (41)
*IKK*	CCTGGAGAACTGCTGTGGAATGAG	ATGGAGGTAGGTCAGAGCCGAAG	Liu et al. (41)
*IκB*	GCTGGTCCATTGCCTCCTGAAC	GTGCCGTCTTCTCGTACAACTGG	Liu et al. (41)
*EF-1α*	AAGCCAGGTATGGTTGTCAACTTT	CGTGGTGCATCTCCACAGACT	Ma et al. (35)

The RNA extraction, cDNA production, and qPCR methods were based on a previous study (34). In brief, total RNA was extracted from the intestine of golden pompano using a HiPure Universal RNA Mini kit (Magen Biotech Co., Ltd., Guangzhou, China) according to the manufacturer’s instructions. The integrity of RNA was assessed using 1% agarose gel electrophoresis, and the concentration was assayed using a Nanodrop 2000 (Thermo Fisher Scientific, MA, USA). We used a PrimeScript™ RT kit (Accurate Biotechnology Co., Ltd., Hunan, China) for reverse-transcription. The gDNA Eraser in the kit eliminated adverse effects. The SYBR^®^ Green Premix Pro Taq HS qPCR Kit (Accurate Biotechnology Co., Ltd., Hunan, China) was used to perform qPCR. qPCR parameters were used as described previously (35, 43). To minimize the impact of incidental factors, each gene was repeated four times and three of the results were selected to calculate target gene mRNA levels using the 2^-ΔΔCT^ method (45).

### *Streptococcus agalactiae* challenge

To complement the impact of cysteine on golden pompano immunity, we conducted an *S. agalactiae* challenge experiment following the feeding trial. The *S. agalactiae* concentration of 2.0 × 10^7^ CFU/fish was the LD50 of golden pompano challenged by *S. agalactiae* for 120 h, as determined previously by our lab (34). After sample collection, we stochastically selected 20 fish per cage with similar size and healthy condition, injected 200 μL of the bacterial suspension at a concentration of 2.0 × 10^7^ CFU/fish into the peritoneal cavity of each fish using a sterile syringe and returned the fish to their respective cages for continued feeding for 120 h. The remaining number of fish in each group was recorded every 12 h, and dead fish were removed. At the end of the challenge trial, all fish were rendered harmless using the alcohol. The same hydrological conditions as those used in the feeding experiment were maintained throughout the challenge. After the challenge experiment, we imported the data into GraphPad Prism 8 software and used the Kaplan-Meier algorithm to calculate the survival curves. A log-rank test was used to compare variances among groups.

### Statistical analyses

Gene expression and serum parameters were analyzed by one-way analysis of variance (ANOVA) using GraphPad Prism 8 and Origin Pro 2021 (OriginLab Corporation, Northampton, MA, USA), respectively. The results of the analysis are presented as mean ± standard deviation (mean ± SD). Tukey’s test was used for multiple comparisons when there was a significant difference (*P<* 0.05).

## Results

### Growth performance

**Figure 1:**
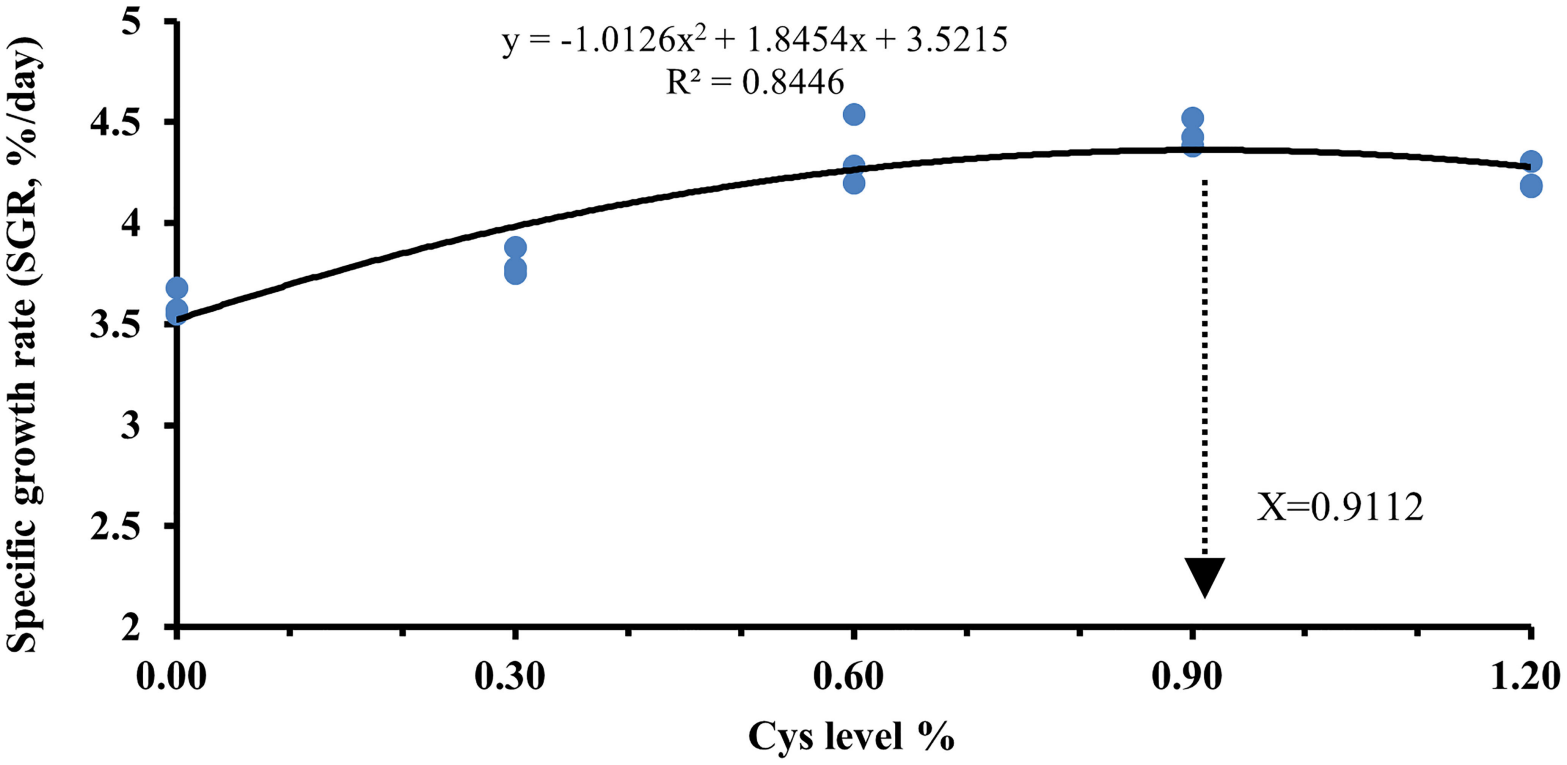
Estimation of the optimal dietary cysteine supplementation level for *T. ovatus* by means of polynomial regression analysis using the SGR.

As shown in **Table 4**, the levels of SR, FI, and CF in C0 fish were not significantly different from those in the other groups (*P* > 0.05). However, growth performance was proportional to cysteine content in the diets. Cysteine markedly increased the levels of FBW, WGR, and SGR in C2, C3, and C4 fish compared to C0 (*P*< 0.05). In addition, dietary cysteine addition of 0.6% was responsible for a significant downregulation of FCR, HSI, and VSI in fish compared with the C0 group (*P*< 0.05). Under these experimental conditions, the optimal cysteine supplementation level in golden pompano diet was 0.91%, according to the polynomial regression results of SGR (**Figure 1**).

**Table 4:** Growth performance of *T. ovatus* fed diets with different dose cysteine supplementation after 8 weeks.

Parameters	Group					*P* valve
	C0	C1	C2	C3	C4	
SR (%)	94.67 ± 1.15^ab^	98.00 ± 2.00^b^	88.67 ± 3.06^a^	89.33 ± 5.03^a^	90.67 ± 2.31^ab^	0.016
IBW (g)	10.17 ± 0.02	10.13 ± 0.05	10.11 ± 0.05	10.15 ± 0.03	10.18 ± 0.04	0.259
FBW (g)	76.00 ± 10.32^a^	85.06 ± 9.87^a^	114.88 ± 14.48^b^	121.36 ± 10.15^b^	107.51 ± 10.11^b^	<0.001
WGR (%)	656.22 ± 34.22^a^	746.32 ± 36.55^a^	1043.06 ± 119.62^b^	1107.58 ± 46.59^b^	969.76 ± 38.55^b^	<0.001
SGR (%/day)	3.60 ± 0.07^a^	3.80 ± 0.07^a^	4.34 ± 0.18^b^	4.44 ± 0.07^b^	4.22 ± 0.07^b^	<0.001
FI (%/day)	1.35 ± 0.04	1.28 ± 0.04	1.27 ± 0.11	1.27 ± 0.05	1.34 ± 0.05	<0.001
FCR (%)	1.98 ± 0.07^b^	1.83 ± 0.07^ab^	1.70 ± 0.17^a^	1.68 ± 0.07^a^	1.82 ± 0.08^ab^	<0.001
HSI (%)	1.04 ± 0.04^b^	0.95 ± 0.03^ab^	0.88 ± 0.04^a^	0.95 ± 0.03^ab^	0.89 ± 0.04^a^	0.017
VSI (%)	6.11 ± 0.26^b^	4.97 ± 0.11^a^	5.19 ± 0.15^a^	5.20 ± 0.21^a^	5.55 ± 0.22^ab^	<0.001
CF (g/cm ^3^)	2.90 ± 0.14^ab^	2.67 ± 0.17^a^	3.15 ± 0.24^ab^	3.52 ± 0.12^b^	2.79 ± 0.12^a^	0.008

values in the same row with different superscripts are significantly different (P< 0.05).

### Serum antioxidant capacity and non-specific immune parameters

**Figure 2:**
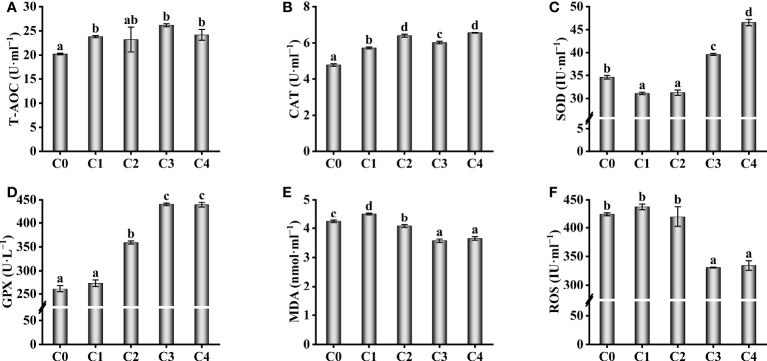
Effect of antioxidant capability such as T-AOC **(A)**, CAT **(B)**, SOD **(C)**, GSH-PX **(D)**, MDA **(E)** and ROS **(F)** in the serum of *T. ovatus* fed diets with different dose cysteine supplementation after 8 weeks. Mean values (n = 9) within values in the picture above with different superscripts are significantly different (*P*< 0.05).

To explore the resistance of fish supplied with exogenous cysteine to oxidative stress, we determined the activity of several antioxidant enzymes in the serum of golden pompano (**Figures 2A–D**). The activity of T-AOC in serum was remarkably increased in C1, C3, and C4 fish compared to C0 fish (*P*< 0.05), but there was no remarkable difference between C2 and C0 fish (*P >*0.05, **Figure 2A**). With higher dietary cysteine levels, CAT activity was significantly higher in all treatment fish than in the C0 fish (*P*< 0.05, **Figure 2B**). Exogenous supplementation with 0.6-1.2% cysteine remarkably upregulated the activities of GSH-PX compared to the C0 fish (*P*< 0.05, **Figure 2D**). Even though 0.3%–0.6% dietary cysteine supplementation decreased the activity of SOD, with higher cysteine supplementation of 0.9%–1.2%, the SOD activity of C3 and C4 fish was considerably higher than in the C0 group (*P*< 0.05, **Figure 2C**). This suggests that the antioxidant effect can only be achieved at a specific dietary cysteine level. This phenomenon was also observed for the levels of MDA and ROS (**Figures 2E, F**). The MDA and ROS levels in the serum of golden pompano decreased considerably when the diet was supplemented with 0.9%–1.2% cysteine, and they were markedly lower than in the C0 group.

**Figure 3:**
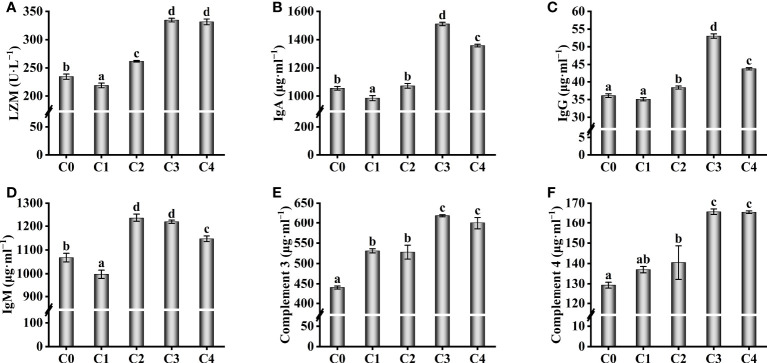
Effect of immunological parameters such as LZM **(A)**, IgA **(B)**, IgG **(C)**, IgM **(D)**, C3 **(E)**, C4 **(F)** in the serum of *T. ovatus* fed diets with different dose cysteine supplementation after 8 weeks. Mean values (n = 9) within values in the picture above with different superscripts are significantly different (*P*< 0.05).

The serum immunological parameters were also affected by exogenous cysteine (**Figure 3**). Exogenous supplementation with 0.3% cysteine did not increase the immunoglobulin (IgM, IgA, and IgG) content and LZM activity in serum of golden pompano, compared to the C0 group (*P* > 0.05, **Figures 3A–D**); however, at 0.6%–1.2% cysteine, the immunoglobulin content and LZM activity were markedly higher than those in the C0 group (*P*< 0.05). The levels of complement 3 and complement 4, important parameters of serum immunology increased significantly with increasing cysteine supplementation (*P*< 0.05), reaching a maximum at 0.9%–1.2% cysteine supplementation (**Figures 3E, F**).

### Midgut histological observation

**Figure 4:**
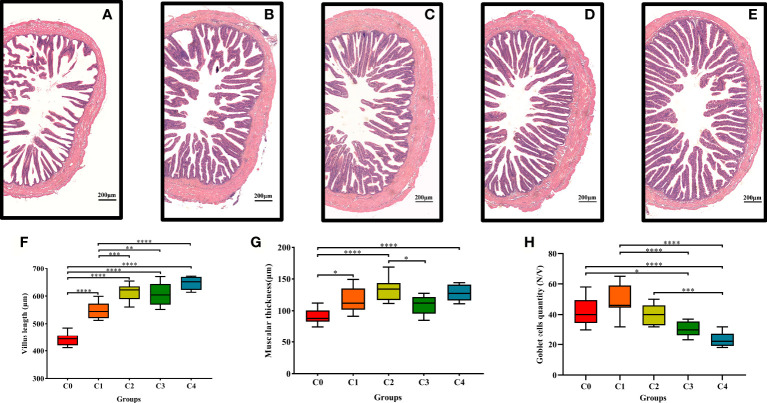
Effects of dietary cysteine on mid-gut morphology of *T. ovatus*. **(A)** 0% cysteine; **(B)**: 0.40% cysteine; **(C)**: 0.80% cysteine; **(D)**: 1.20% cysteine; **(E)**: 1.60% cysteine. Scale bar: 200 μm. The villus length **(F)**, muscular thickness **(G)**, and goblet cells quantity **(H)** of mid-gut in *T. ovatus.* data are presented as mean ± SD (n = 9). Asterisks *, **, ***, and **** indicate statistically significant difference between treated group and control group at *P*< 0.05, *P*< 0.01 *P*< 0.001, and *P*< 0.0001, respectively.

The histological parameters of the golden pompano intestine are shown in **Figure 4**. With higher cysteine supplementation, intestinal villus length and muscular thickness increased markedly (**Figure 4F**). Villus length was significantly higher in C2, C3, and C4 fish than in C0 and C1 fish (*P*< 0.05). In addition, the statistical results indicated that exogenous supplementation with 0.3% and 1.2% cysteine substantially increased muscular thickness, compared to C0 fish (*P*< 0.05, **Figure 4G**). Nevertheless, cysteine supplementation decreased the number of goblet cells per intestinal villus (**Figure 4H**). The abundance of goblet cells was considerably lower in C3 and C4 fish compared to that in the controls (*P*< 0.05).

### 16S rDNA high-throughput sequencing of the intestinal flora

**Figure 5:**
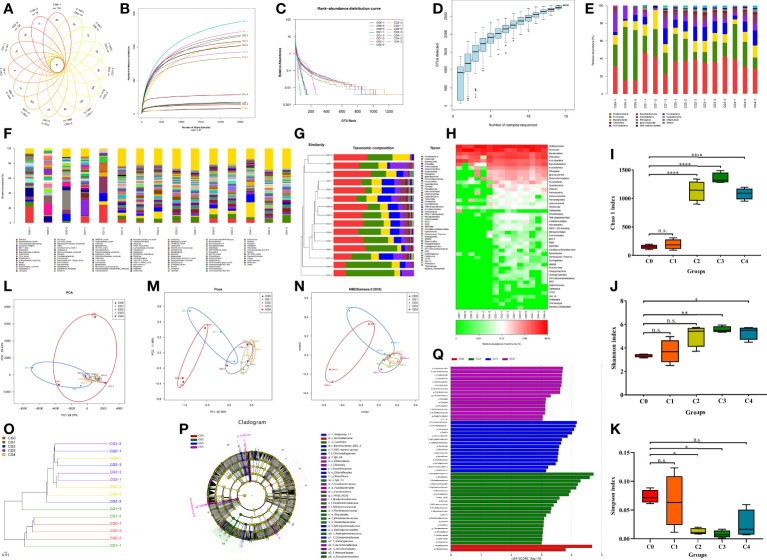
Cysteine supplementation regulated gut microbiota composition and abundance. Profile of the intestinal microbiota (n = 3). The basic structure of the gut microbiota was determined by Venn diagram **(A)**, Rarefaction curves **(B)**, Rank-Abundance curves **(C)**, species accumulation curves **(D)**, relative abundance histograms of phylum **(E)** and genus **(F)**, Mcrobial community barplot with cluster tree **(G)**, and heat map comparison **(H)** were depicted. The diversity and richness of the communities were compared by alpha diversity indices, including Chao 1 index **(I)**, Shannon index **(J)**, and Simpson index **(K)**. The community diversity and richness were compared by Principal Component Analysis **(L)**, UniFrac-based principal co-ordinates analysis **(M)**, and UniFrac-based non-metric multi-dimensional scaling analysis **(N)** were performed for β-diversity analysis. Communities or species with significantly different effects on sample delineation were identified by UniFrac-based cluster tree **(O)**, Evolutionary branching plots **(P)**, and Linear discriminant analysis **(Q)**. "n.s."indicate No significant difference. Asterisks *, **, and **** indicate statistically significant difference between treated group and control group at *P* < 0.05, *P* < 0.01, and *P* < 0.0001, respectively.

We obtained 678,797 clean reads and 2,807 OTUs by high-throughput sequencing of the intestinal microbes of the golden pompano. A Venn diagram was produced to visualize that 15 OTUs were identical in all groups, and dietary cysteine increased the amount of specific OTUs in the gut flora (**Figure 5A**). Rarefaction curves (**Figure 5B**), OTU rank-abundance curves (**Figure 5C**), and species accumulation curves (**Figure 5D**) converged to saturation, allowing assessment of sequencing depth, species evenness, and species richness. The dominant phyla included Proteobacteria, Firmicutes, and Bacteroidetes (**Figure 5E**). Firmicutes showed a decreasing trend (0%–0.9%) and then increased (0.9%–1.2%) with increasing dietary cysteine content. At the genus level, *Ralstonia*, *Saccharibacterianorank*, and *[Ruminococcus] gnavus* were dominant (**Figure 5F**). A sample clustering tree (**Figure 5G**) and abundance similarity clustering (**Figure 5H**) of intestinal flora among the groups showed that cysteine supplementation enriched the composition of the intestinal flora of golden pompano. With increasing dietary cysteine content, the abundance of the intestinal flora of golden pompano gradually increased.

To further investigate the effect of cysteine on the abundance and diversity of intestinal microbial populations, we used alpha diversity index analysis, which showed that supplementation with 0.6%–1.2% cysteine significantly increased the Chao1 index (**Figure 5I**) and reduced the Simpson index (**Figure 5K**), compared to the controls, except for the C0 and C4 fish in which the Simpson index did not differ significantly. Similarly, the Shannon index showed (**Figure 5J**) that the exogenous supplementation with 0.9%–1.2% cysteine increased the abundance and diversity of the flora.

We used PCA, UniFrac-based PCOA, and UniFrac-based NMDS for comparative analysis of the similarity between groups and samples (β diversity analysis). Exogenous supplementation with 0.6%–1.2% cysteine caused a similar gut microbial composition across experimental fish, which differed from that of the C0 fish (**Figures 5L, N**). The UniFrac-based PCOA weighted results showed that differences in the PC1 axis and PC2 axis explained 62.56% and 11.46% of the variation, respectively (**Figure 5M**). UniFrac-based cluster tree analysis using the unweighted pair group method with arithmetic mean was used to visualize the evolutionary similarities and differences of microorganisms in different samples, and the results showed that the gut flora of C0 and C1 fish were more similar and clustered in one branch. In contrast, C2, C3, and C4 fish were similar and clustered on a different branch (**Figure 5O**).

To identify communities or species that had a significant differential effect on sample delineation, we used the non-parametric factorial Kruskal-Wallis sum-rank test method to detect characteristics with significant abundance differences and to identify taxa that differed significantly in abundance. Linear discriminant analysis was applied using LEfSe software to estimate the magnitude of the effect of abundance on the different effects for each component (species). Evolutionary branching plots (**Figure 5P**) and linear discriminant analysis (**Figure 5Q**) showed that only the class Actinobacteria and the genus *Megasphaera* played a significant role in C0 fish. In contrast, C2, C3, and C4 fish had more diverse gut flora. Six phyla (Proteobacteria, Bacteroidetes, Actinobacteria, Firmicutes, Chloroflexi, and Ignavibacteriae), nine orders, eleven families, and eight genera were present more frequently in C2 fish. Four phyla (Proteobacteria, Acidobacteria, Chloroflexi, and Bacteroidetes), six orders, four families, and six genera were more abundant in the C3 fish. Four phyla (Fusobacteria, Proteobacteria, Firmicutes, and Chlorobi), five orders, five phyla, six families, and four genera were more abundant in C4 fish.

### Antioxidant enzyme expression in the intestine

**Figure 6:**
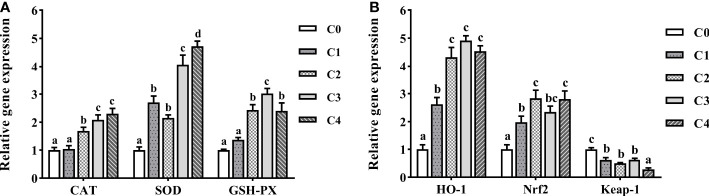
The expression profiles of antioxidant genes **(A)** and signaling pathway **(B)** in the intestine of *T. ovatus* fed diets with different dose cysteine supplementation after 8 weeks. Mean values (n = 9) within values in the picture above with different superscripts are significantly different (*P*< 0.05).

We examined the expression of antioxidant enzyme genes in the intestine of golden pompano to examine the effects of exogenous cysteine on antioxidant stress capacity (**Figure 6A**). Compared to the control group, *CAT* and *GSH-PX* expression were substantially upregulated in C2, C3, and C4 fish (*P*< 0.05), and the *GSH-PX* expression was highest in the C3 group. The CAT expression levels between the C3 and C4 groups were not markedly different (*P* > 0.05), with both significantly higher than the other groups (*P*< 0.05). The effect of exogenous cysteine on *SOD* expression was considered significant. The expression of these three genes was upregulated owing to exogenous cysteine supplementation, compared to that in the control group, and it was considerably higher than that in the C0 group (*P*< 0.05). In addition, dietary cysteine reduced intestinal *Keap-1* mRNA levels and led to upregulation of *HO-1* and *Nrf2* (**Figure 6B**).

### Intestine immunity-related gene expression analysis

**Figure 7:**
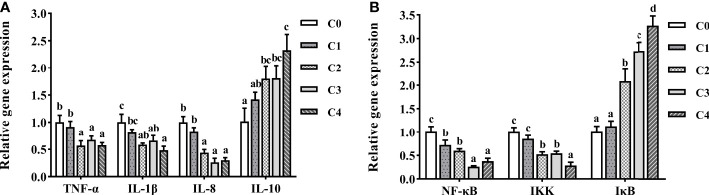
The expression profiles of inflammatory genes **(A)** and signaling pathway **(B)** in the intestine of *T. ovatus* fed diets with different dose cysteine supplementation after 8 weeks. Mean values (n = 9) within values in the picture above with different superscripts are significantly different (*P*< 0.05).

To gain further information on the effect of exogenous cysteine on intestine immunity of golden pompano, we selected the essential genes of inflammatory response-related cytokines and the NF-κB pathway for qPCR (**Figure 7**). Exogenous supplementation with cysteine suppressed the expression of *TNF-α*, *IL-1β*, *IL-8* (**Figure 7A**), *NF-κB*, and *IKK* (**Figure 7B**) in the intestine of golden pompano. Dietary cysteine supplementation of 0.6%–1.2% led to lower expression levels of *TNF-α*, *IL-1β*, *NF-κB*, *IKK*, and *IL-8*, compared to C0 fish (*P*< 0.05). Meanwhile, expression of *IκB* (**Figure 7B**) and *IL-10* (**Figure 7A**) was significantly upregulated by exogenous cysteine, compared with that in the controls (*P*< 0.05). This demonstrated that cysteine could promote intestine immunity in golden pompano.

### *Streptococcus agalactiae* challenge

**Figure 8:**
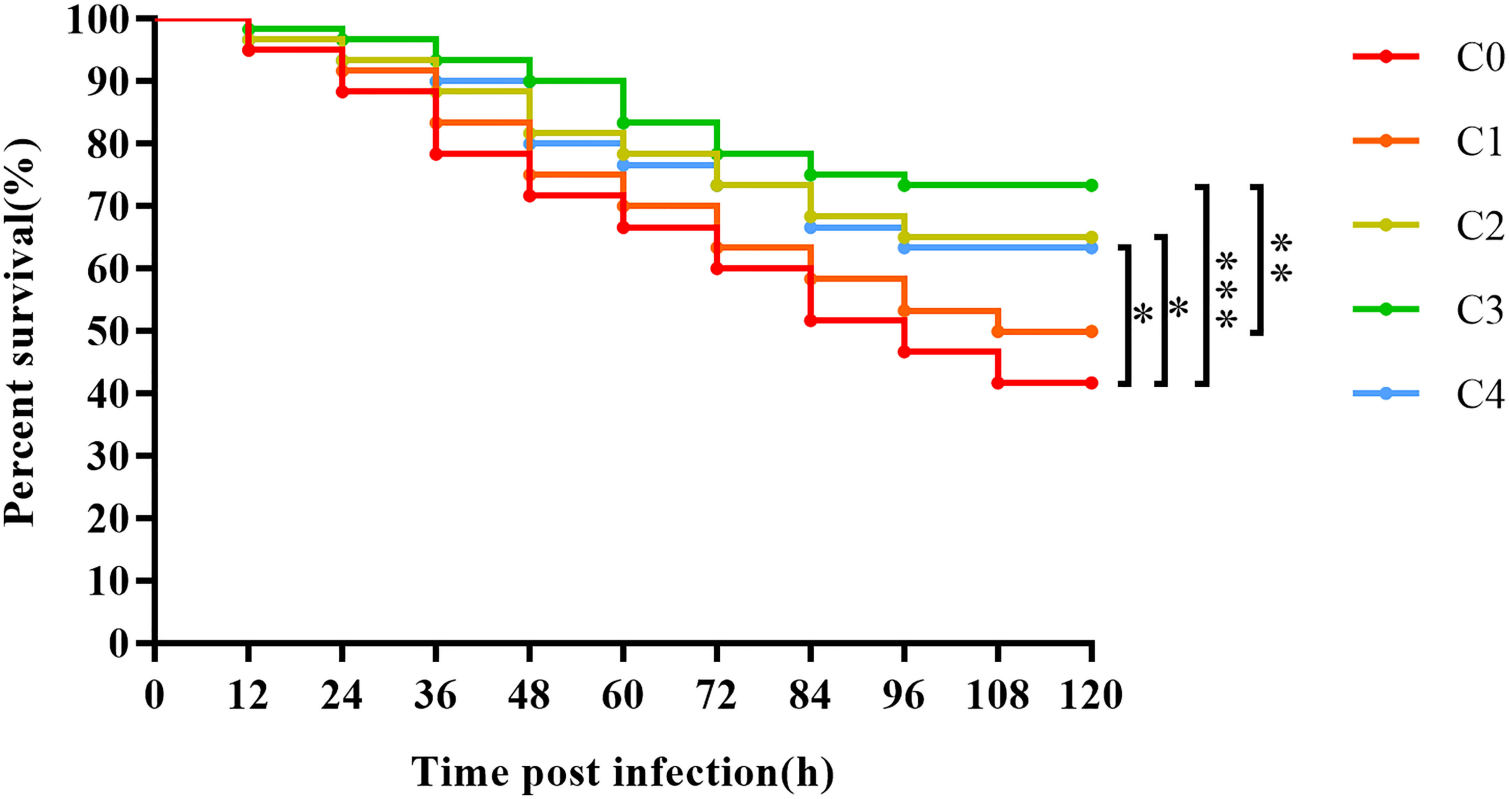
The Kaplan-Meier survival analysis of *T. ovatus* after *S. agalactiae* infection. Asterisks *, **, and *** indicate statistically significant difference between the two groups at *P* < 0.05, *P* < 0.01, and *P* < 0.001, respectively.

The survival rates of juvenile golden pompano after *S. agalactiae* challenge is presented in **Figure 8**. The survival rates of C0 (0), C1 (0.3%), C2 (0.6%), C3 (0.9%), and C4 (1.2%) fish after the challenge for 120 h were 41.67%, 50.00%, 63.33%, 73.33%, and 63.33%, respectively. The survival of juvenile golden pompano gradually increased with an increase in dietary cysteine supplementation from 0% to 0.9%. Exogenous supplementation with 0.6%–1.2% cysteine markedly improved the resistance of golden pompano to *S. agalactiae*, compared to the C0 group (*P*< 0.05). Thus, appropriate cysteine supplementation can greatly promote immunity in golden pompano.

## Discussion

### Supplementation of low-fishmeal diets with cysteine promotes growth of juvenile golden pompano

In the present study, supplementation with 0.6%–1.2% cysteine remarkably increased the FBW, WGR, and SGR levels in golden pompano. Moreover, adding 0.6% cysteine to the diet decreased the FCR, HSI, and VSI significantly, compared to the C0 group. These indices indicated that dietary supplementation with cysteine greatly enhanced the growth performance of golden pompano. Similar findings were made in a previous study on *Psetta maxima*, which showed that a moderate amount of dietary cysteine (0.3%) enhanced the SGR levels and the mean final weight of *P. maxima* and improved its growth performance (20). Oxidative stress and immune capacity reduction caused by low fishmeal content are the main reasons for reduced growth performance (8, 9, 46). In the present study, there were many reasons that cysteine supplementation improved the growth performance of golden pompano in low fishmeal conditions, such as the increased abundance and diversity of gut microbes (31), increased antioxidant capacity (47, 48), and improved immune ability (27). Our results showed that dietary supplementation with 0.6%–1.2% cysteine could increase the activity of serum antioxidant enzymes (T-AOC, CAT, and GSH-PX) and decrease the serum MDA and ROS levels in juvenile golden pompano. Meanwhile, non-specific serum immune parameters were improved, and the levels of immunoglobulins (IgM, IgA, and IgG), complement 3, complement 4, and LZM activity were increased, which may help the fish cope with oxidative stress and immunity reduction caused by low-fishmeal diets, thus improving growth performance. Our findings are similar to those found in *Seriola lalandi* (47) and *Scophthalmus maximus* L (48), where cysteine enhanced growth performance by improving resistance to oxidative stress and intestine immunity.

### Supplementation of low-fishmeal diets with cysteine improves the intestinal physical barrier in juvenile golden pompano

The intestinal physical barrier is a critical component of intestine immunity with the function of taking in nutrients and defending against harmful substances (49, 50). Low-fishmeal diets damage the intestinal physical barrier; for example, replacing fishmeal with soybean meal reduces the villi length of *Epinephelus coioides* (12), *Litopenaeus vannamei* (49), and *Penaeus monodon* (50) and damages the intestinal mucosal folds, leading to intestinal inflammation. Cysteine effectively protects the physical barrier of the intestine. For instance, cysteine protects the intestine of broiler chicks by increasing the length of the intestinal villi (51). In addition, cysteine has been found to improve intestinal integrity and permeability and reduce the intestinal inflammatory response in pigs (25, 27). Similarly, our results showed that exogenous cysteine supplementation at 0.6% and 1.2% greatly improved intestinal villus length and muscular thickness, thus effectively alleviating the weakening of the intestinal physical barrier associated with low-fishmeal diets. Alterations in the intestinal physical barrier typically accompany changes in the abundance of intestinal flora (52, 53) and expression of intestinal immune-related genes (54). Therefore, we propose that the protective effect of cysteine on the intestinal physical barrier of golden pompano may be correlated with increased abundance of intestinal flora and high expression of intestinal antioxidant stress- and immune-related genes.

### Supplementation of low-fishmeal diets with cysteine improves the diversity of intestinal flora in juvenile golden pompano

Maintaining the diversity and abundance of intestinal flora is essential for the intestinal health of fish, and disturbances to the intestinal flora tend to damage the physical barrier of the intestine and elicit inflammatory responses (53, 55). In our study, alpha and beta diversity analysis of the intestinal flora showed that supplementation of the diet with cysteine regulated the abundance and diversity and improved the structure of intestinal flora in golden pompano. Alpha diversity results demonstrated that exogenous supplementation with 0.6%–1.2% cysteine remarkably increased the Chao 1 index and decreased the Simpson index, thus the diversity of the intestinal flora was markedly improved, and similar results were found regarding the Shannon index (C2 and C3 fish). At the phylum level, the dominant taxa included Proteobacteria, Firmicutes, Bacteroidetes, Fusobacteria, Chloroflexi, and Actinobacteria. Appropriate addition of cysteine to the diet increased the relative abundance of Proteobacteria and Bacteroidetes and decreased the relative abundance of Firmicutes. Bacteroidetes are essential players in polysaccharide (56–58) and cholesterol metabolism (59), and it is possible that altering the abundance of Bacteroidetes is one of the ways cysteine regulates nutrient metabolism. At the genus level, *Ralstonia*, *Saccharibacterianorank*, and *[Ruminococcus] gnavus group* were predominant in the intestinal tract of golden pompano. Adequate cysteine addition can thus increase the relative abundance of the *[Ruminococcus] gnavus* group. Similarly, previous studies have shown that dietary cysteine has a positive effect on intestinal flora richness and diversity in gestating sows (31) and *S. maximus* L (48).

### Supplementation of low-fishmeal diets with cysteine enhances the expression of intestinal antioxidant and immune-related genes in juvenile golden pompano

The protective effect of cysteine on intestinal integrity may be related to signaling pathways associated with antioxidant and inflammatory responses. Numerous studies confirmed that excessive substitution of fishmeal in diets elicits oxidative stress in the fish intestine owing to excessive anti-nutrients, increasing ROS levels, and leading to structural damage in the intestine (8, 9). For instance, the addition of plant proteins to diets causes oxidative stress and structural damage in the intestine of *S. maximus* L *(* 9 *).*, *Monopterus albus* (11), and *Amurenser schrenckii* (60). ROS levels are predominantly regulated by the Nrf2/Keap-1 signaling pathway (43, 61). Nrf2 can reduce ROS levels and increase the expression of antioxidant enzyme genes in golden pompano by enabling the high expression of *HO-1* (42). Our results indicate that cysteine supplementation activated the Nrf2/Keap-1 signaling pathway in the intestine of golden pompano. Downregulation of *Keap-1* mRNA levels led to the upregulation of *HO-1* and *Nrf2* mRNA levels, increasing the mRNA levels of intestinal antioxidant enzymes (*CAT*, *SOD*, and *GSH-PX*) and the corresponding enzyme activities in serum.

Oxidative stress not only damages tissue integrity but may also elicit inflammatory responses and reduce the immune capacity of the body (41). Inflammatory responses are regulated by a combination of anti-inflammatory cytokines (IL-10, TGF-β, etc.) (30, 62) and pro-inflammatory cytokines (IL-1β, IL-8, TNF-α, etc.) (30, 48, 63), which in turn are typically modulated through the NF-κB/IκB/IKK signaling pathway (64, 65). A study showed that cysteine inhibited *NF-κB* expression and downregulated pro-inflammatory cytokines (*IL-6*, *TNF-α*, and *IL-8*) in the intestine of piglets, which improved the physical barrier of the intestine and promoted intestinal health (25). Similarly, we found that *NF-κB*, *IKK* (**Figure 7B**), *IL-1β*, *IL-8*, and *TNF-α* (**Figure 7A**) in the intestine of golden pompano were negatively correlated with dietary cysteine content, whereas the expression levels of *IκB* and *IL-10* showed contrasting patterns. These results suggest that dietary supplementation with 0.6%–1.2% cysteine (C2, C3, and C4 fish) decreases the expression level of pro-inflammatory cytokines by inhibiting the NF-κB signaling pathway. Thus, the inflammatory response was suppressed, and the intestine immunity of golden pompano was improved.

### Supplementation of low-fishmeal diets with cysteine enhances the resilience of juvenile golden pompano to *Streptococcus agalactiae*

Infectious diseases caused by *S. agalactiae* have frequently erupted in recent years in large-scale aquacultures of marine fish because of high culture densities (34). Therefore, we also examined the protective effect of cysteine during an *S. agalactiae* challenge in golden pompano. Survival curves showed that cysteine had a positive effect on the survival rate of golden pompano. The survival rate was remarkably higher in C2, C3, and C4 (0.6%–1.2% cysteine) than in C0 fish. Bacterial challenge can reduce the diversity of intestinal flora in fish (66, 67), whereas our results showed that cysteine substantially increased the diversity of the intestinal flora. Therefore, considering the high agreement between the survival rate of the present challenge test and intestinal immune parameters, such as histological results of the intestine, results of microbial diversity analysis, and intestinal immune gene expression, we infer that cysteine may have enhanced the resistance of golden pompano to *S. agalactiae* by improving its intestine immunity.

## Conclusions

Overall, a moderate level of cysteine (0.6%–1.2%) improved the growth performance of golden pompano, and the optimum dietary cysteine supplementation level for juvenile golden pompano was 0.91%, based on polynomial regression analysis of SGR. Moderate dietary cysteine also improved the abundance and diversity of intestinal flora and enhanced the structural integrity of the intestine to maintain the stability of the intestinal physical barrier. Furthermore, dietary cysteine activated the Nrf2/Keap1/HO-1 signaling pathway and inhibited NF-κB signaling, increased intestinal antioxidant enzyme genes (*CAT*, *SOD*, and *GSH-PX*) and anti-inflammatory cytokine mRNA levels, increased serum antioxidant enzyme activity, and substantially improved intestine immunity, resulting in higher survival rates of golden pompano exposed to *S. agalactiae*. These findings provide new insights into the development and use of cysteine as a dietary supplement.

## Data availability statement

The data presented in the study are deposited in the NCBI repository, accession number: PRJNA889580. The data can be found below: https://www.ncbi.nlm.nih.gov/search/all/?term=PRJNA889580.

## Ethics statement

The animal study was reviewed and approved by Animal Care and Use Committee of the South China Sea Fisheries Research Institute of the Chinese Academy of Fishery Sciences.

## Author contributions

D-CZ designed the experiments and wrote the manuscript. J-XL conducted the experiments and wrote the manuscript. K-CZ: Methodology, Software. H-YG: Data curation, Methodology. B-SL: Supervision, Software. NZ: Visualization, Investigation. All authors contributed to the article and approved the submitted version.

## Funding

This research was financially supported by Hainan Yazhou Bay Seed Lab (B21HJ0702), China Agriculture Research System of MOF and MARA (CARS-47), Central Public-Interest Scientific Institution Basal Research Fund of South China Sea Fisheries Research Institute CAFS (2021SD12), Central Public-interest Scientific Institution Basal Research Fund, CAFS (NO.2020TD29), Key Projects of Joint Fund for Regional Innovation and Development of NSFC (U20A2064), National Marine Genetic Resource Center, Guangdong Provincial Special Fund for Modern Agriculture Industry Technology Innovation Teams (2019KJ143).

## Conflict of interest

The authors declare that the research was conducted in the absence of any commercial or financial relationships that could be construed as a potential conflict of interest.

## Publisher’s note

All claims expressed in this article are solely those of the authors and do not necessarily represent those of their affiliated organizations, or those of the publisher, the editors and the reviewers. Any product that may be evaluated in this article, or claim that may be made by its manufacturer, is not guaranteed or endorsed by the publisher.

## Abbreviations

T-AOC, total antioxidant capacity; CAT, catalase; GSH-PX, glutathione peroxidase; SOD, superoxide dismutase; MDA, malondialdehyde; ROS, reactive oxygen species; LZM, lysozyme; IgA, immunoglobulins A; IgG, immunoglobulins G; IgM, immunoglobulins M; Nrf2, nf-e2-related nuclear factor 2; Keap-1, Kelch-like ECH-associated protein-1; HO-1, heme oxygenase-1; NF-κB, nuclear factor kappa B; IκB, inhibitor protein-κB; IKK, IκB kinase; TNF-α, tumor necrosis factor-α; IL-1β, interleukin 1β; IL-8, interleukin 8; IL-10, interleukin 10; WGR, weight gain rate; SGR, specific growth rate; FCR, feed conversion ratio; CF, condition factor; SR, survival rate; HSI, hepatosomatic index; VSI, viscerasomatic index; FI, feed intake; IBW, initial body weight; FBW, final body weight; FBL, final body length; DDI, dry diet intake; NOD, number of days; VW, viscera weight; LW, liver weight; NWG, net weight gain; FFN, final fish numbers.
